# Association of Non-Dipping Blood Pressure Patterns with Diabetic Peripheral Neuropathy: A Cross-Sectional Study among a Population with Diabetes in Greece

**DOI:** 10.3390/nu15010072

**Published:** 2022-12-23

**Authors:** Styliani Ntavidi, Panagiota Katsanou, George Marakomichelakis, Maria-Iosifina Kasdagli, Eleni Antiochou, Ioulia Mpali, Anda-Monica Kakou, Konstantinos Tsioufis, George Dimitriadis, Vaia Lambadiari

**Affiliations:** 1 Outpatient Clinic of Diabetic Foot, 4th Department of Internal Medicine, General Hospital of Evangelismos, 10676 Athens, Attiki, Greece; 2 Department of Hygiene, Epidemiology and Medical Statistics, National and Kapodistrian University of Athens, 11527 Athens, Attiki, Greece; 3 Department of Cardiology, General Hospital of Hipokration, 11527 Athens, Greece; 4 2nd Department of Internal Medicine, Research Institute and Diabetes Center, National and Kapodistrian University of Athens Medical School, Attikon University Hospital, 12462 Haidari, Greece

**Keywords:** diabetic neuropathy, peripheral neuropathy, diabetic foot, hypertension, dipping, insulin resistance, hyperinsulinemia

## Abstract

Diabetic peripheral neuropathy (DPN) is present in 20–50% of cases with diabetes. The pathophysiology of DPN is not yet clear regarding hypertension (HTN). The aim of this study was to assess the association between the stages of DPN and HTN in a Greek population with diabetes. We examined 102 adults for diabetic neuropathy (DPN) from November 2020 to December 2021, using the Toronto Clinical Neuropathy Scale System (TCNSS) to categorize them into two groups (no/mild DPN versus medium/severe DPN). Ambulatory blood pressure monitoring was performed to evaluate their hypertensive status. Univariate and multivariate logistic regression analyses were performed to assess the association between the stage of DPN and HTN. The multivariate analysis, considering sex, age, and dipping status, did not show statistically significant associations between stages of HTN and DPN. However, in contrast to dippers, non-dippers had an almost four-times higher risk of developing medium-to-severe DPN (odds ratio (OR) 3.93; 95% confidence interval (CI) [1.33–11.64]); females, in contrast to males, had a 65% lower risk of developing moderate/severe DPN (OR 0.35; 95%CI [0.14–0.92]). In conclusion, our findings showed no statistically significant associations between DPN and HTN; however, dipping status, hyperglycemia, and female sex were shown to play a role in the pathophysiology of DPN.

## 1. Introduction

Diabetic complications of the lower extremities are common [1]. Diabetic peripheral neuropathy (DPN) is the most common neuropathy in developed countries and worldwide [1,2]. According to the International Diabetes Federation (IDF), the prevalence of DPN is expected to rise from 40–60 million people in 2020 to 578 million by 2030 and to 700 million by 2045 [3]. Up to 50% of populations with diabetes may develop DPN during their lifetime [4,5,6], while it is estimated that subjects with type 2 diabetes (T2DM) have a two-fold higher risk compared to type 1 diabetes (T1DM) [7,8,9].

Diabetic complications of the lower extremities are also costly [10]. Diabetic foot, a combination of DPN and ulceration in 85% of cases, is the main reason behind non-traumatic amputations of the lower limbs [10]. It is estimated that every 30 s, one amputation happens in the diabetic population [10]. This leads to almost 24.4% of the Total Fund of Global Health being spent on complications of the limbs [11]. Furthermore, the psychological burden from neuropathic pain and impaired sensibility adds to the reduced quality of life, which may lead to depression [1].

Finally, the pathogenic mechanisms of diabetic complications, including foot complications, are also complex and are due to vascular damage. It is well known that the underlying drivers of the development of microvascular/macrovascular disease are insulin resistance and tissue exposure to chronic hyperglycemia and hyperlipidemia (glucotoxicity/ lipotoxicity) [12,13]. While DPN is often routinely considered a microvascular complication of diabetes, this concept does not fully address the complexities of direct neuronal involvement [1,14].

Identifying the risk factors of diabetic complications may enable health providers to set up better prevention strategies to alleviate the personal, social, and economic burden of society. Several studies have assessed the role of hypertension (HTN) in the development of DPN but with inconsistent results. Therefore, the aim of this study was to assess the association between the stage of DPN and HTN in a Greek diabetic population.

## 2. Materials and Methods

We conducted an observational study from November 2020 to December 2021 in Evangelismos General Hospital in Athens, Greece. We registered 102 patients with diabetes from the “Outpatient Clinic of Diabetic Foot” and the “Outpatient Clinic of Hypertension”. The study protocol was approved by the Scientific and Ethics Committee of Evangelismos General Hospital. The patients were informed in detail about the purpose of the study and signed a consent form.

A qualified and trained physician filled out a personal structured sheet for every participant, with detailed information on demographic characteristics and medical history, with an emphasis on the type and duration of diabetes (DM), hypertension (HTN) and dyslipidemia, medications (including hypoglycemic/antihypertensive/lipid-lowering agents), body mass index (BMI), smoking and alcohol use, and physical activity/exercise. In all participants, blood test analysis was conducted in the early morning hours (08:00–10:00 am) after an overnight fast for at least 10 h. The eGFR was calculated according to the MDRD equation.

Blood pressure (BP) was measured by the ambulatory blood pressure monitoring (ABPM) device from Micro life. The function of this device was explained in detail to each participant to obtain cooperation. With an appropriately sized BP cuff (medium, large, x-large) on the non-dominant upper limb and a frequency of measurements of every 20 min, the participants were encouraged to maintain their usual schedule and to record sleep hours during the day and night.

All participants underwent screening for peripheral neuropathologic symptoms, including numbness of the lower limbs, acupuncture-like sensation, fatigue, staggered walking, and upper limb symptoms. All assessments were performed by the same physician. The symptoms were graded according to the standard criteria for the evaluation of peripheral neuropathy (including acupuncture pain, touch sensation, temperature sensation, vibration sensation, positional sensation, bilateral knee reflex, and ankle reflex).

For the classification of hypertension (HTN), we followed the ESH/ESC guidelines (2018 ESC/ESH Clinical Practice Guidelines for the Management of Arterial Hypertension) [15]. More specifically, optimal BP was defined as systolic blood pressure (SBP) <120 mmHg and diastolic blood pressure (DBP) <80 mmHg, normal SBP as 120–129 mmHg and DBP 80–84 mmHg, high normal SBP as 130–139 mmHg and DBP 85–89 mmHg, grade 1 HTN as 140–159 mmHg/90–99 mmHg, and grade 2 HTN as 160–179 mmHg/100–109 mmHg. Dipping was estimated as an abnormal fall in mean SBP during sleep up to 10–20%. Dipping of less than 10% of the mean daytime SBP was considered non-dipping, whereas a rise in the nighttime mean SBP was considered reverse dipping.

Using the 2002 Toronto Clinical Neuropathy Scoring System (TCNSS), which includes a neurologic score, neurologic reflex score, and sensory function score, DPN was graded as follows: (1) 0–5 points, no evidence of DPN (non-DPN); (2) 6–8 points, mild DPN; (3) 9–11 points, moderate DPN; and (4) 12–19 points, severe DPN. Participants were divided into two subgroups, one with absent/mild DPN and the other with moderate/severe DPN.

### Statistical Analysis

Participants’ data were summarized as the mean and standard deviation for the continuous variables and as frequencies and percentages for categorical variables. For continuous variables, differences between the two groups (those with absent/mild DPN versus those with moderate/severe DPN) were assessed via a two-sample *t*-test. For categorical variables, relevant differences were assessed by Pearson’s chi-square test. Initially, we performed univariate logistic regression models and afterward a multivariate logistic regression model to evaluate the association between the stage of DPN and that of HTN, using the backward stepwise approach. In the final logistic regression model, only the statistically significant variables were included (*p* <0.05), whereas age, sex, and stages of hypertension were included as potential confounders. Statistical analysis of the data was performed using Stata/SE 14.0.

## 3. Results

**Figure 1:**
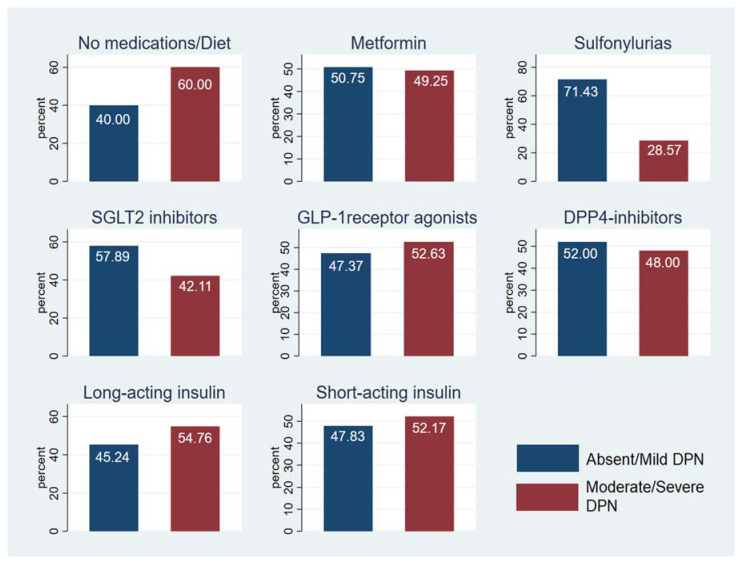
Schematic association of antidiabetic medication and DPN status.

**Figure 2:**
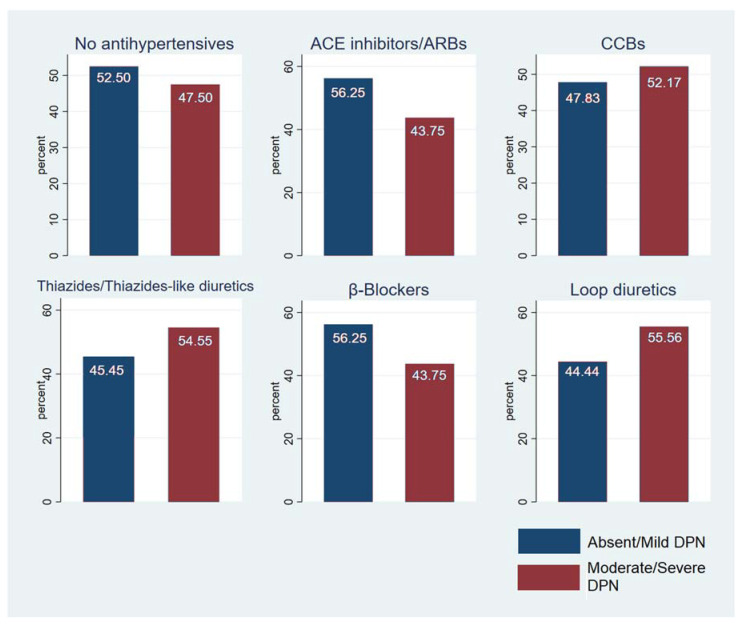
Schematic presentation of antihypertensive therapy and DPN status.

**Figure 3:**
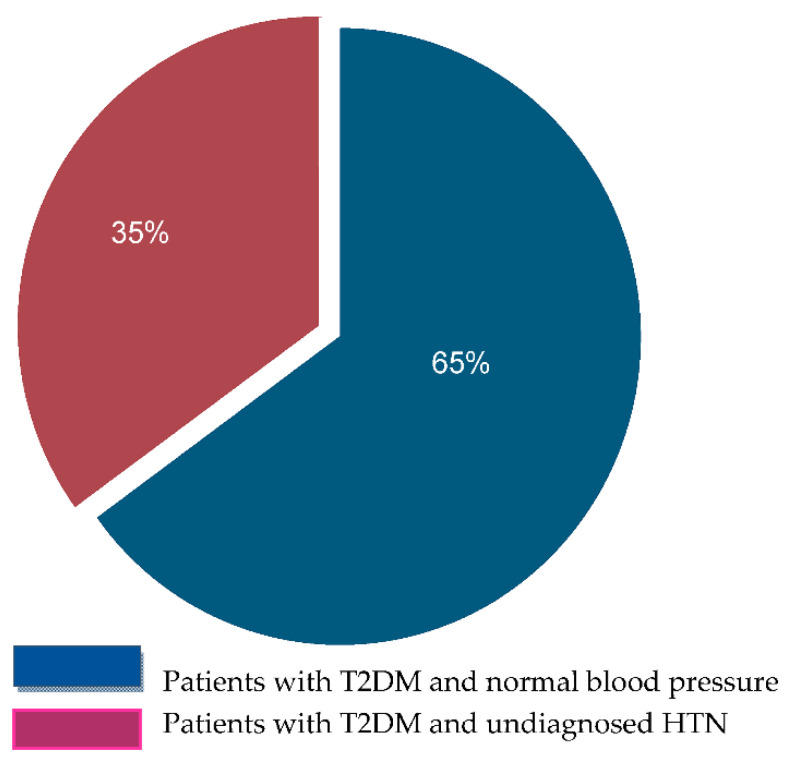
Individuals with T2DM under no antihypertensive medication.

Most of our patients were male (64%), and the mean age was 60.45 ± 11.3 years. The mean 24 h SBP and DBP were 123.47± 11.92 mmHg and 71.23 ± 7.45 mmHg, respectively (Table 1). Most participants had T2DM (94.11%) and 44% were obese. Of the individuals with T2DM, 50 were on oral antidiabetic agents (49.02%), 18 were on insulin (17.65%), and 30 (29.41%) were on a combination of insulin and oral agents (Figure 1). The majority were receiving antihypertensive therapy (78.43%) and specifically receiving ACE inhibitors or ARBs (Figure 2). Forty patients were receiving no antihypertensive therapy and, independently of DPN status, 35% of them were found with unknown HTN (Figure 3). The 24 h mean SBP was 123.47 ± 11.92 mmHg. The 24 h mean DBP and nocturnal SBP were found to be statistically significantly associated with DPN status (*p*-value 0.0181 and 0.0371, respectively; Table 2, and 62.5% of the participants were either non-dippers or reverse dippers (Table 3). The two groups differed in HbA1c, 24 h DBP, nighttime SBP, nighttime DBP, and BMI (Table 1, Table 2 and Table 3). Smoking status and the number and type of antihypertensives and antidiabetics were not found to be statistically significant when associated with the stage of DPN or dipping status (data not shown).

To investigate the relationship between the stages of DPN and HTN, we performed univariate and multivariate logistic regression analyses (Table 4). The multivariate model included other possible independent confounders, such as age, sex, ABPM dipping, and 24 h hypertension stages, and showed that females had a lower risk of moderate or severe DPN (OR 0.35; 95%CI [0.14–0.92]) versus male participants (Table 4). Furthermore, non-dippers had a higher risk of moderate/severe DPN (versus dippers) (OR 3.93; 95%CI [1.33–11.64], Table 4), while age and 24 h-hypertension stage did not reach statistical significance in the multivariate model.

## 4. Discussion

In our study, we evaluated the relationship between the stages of DPN and HTN in a Greek population with diabetes (93% T2DΜ, 7% T1DΜ). Moderate/severe DPN was present in 89.6% and 10.4% of T2DΜ and T1DΜ participants, respectively. Our results also showed that, in contrast to men with T2DM, women have a lower risk of developing DPN. In agreement with other studies, such as by Rossboth et al. [16], this may be explained by the fact that the majority of men with T2DM lack adherence to antidiabetic therapy, resist lifestyle changes and the cessation of smoking, and are more vulnerable to minor trauma of the feet. On the contrary, Khan et al. [17] showed that the female gender is more susceptible to DPN progression. This heterogeneity in results may be due to racial and cultural differences.

Several studies have shown an association between HTN and DPN [18,19,20,21,22]. Cardoso et al. [23] and Gebabo et al. [24] reported that patients with resistant HTN had a 2.36-fold higher risk of developing DPN. Likewise, Huang et al. showed that normotensive individuals with diabetes and DPN had a higher normal SBP [25]. These studies suggest that the existence of DPN may be associated with a higher BP in normotensives and with a more difficult-to-control HTN in hypertensives.

Blood pressure is regulated by many factors and, therefore, its management becomes difficult when the severity of these factors increases despite the use of antihypertensives.

The risk factors of hypertension in general and particularly in patients with DM include, among others, older age, lifestyle (obesity, excessive salt intake, less physical activity/exercise, smoking, alcohol consumption, mental stress), hyperglycemia, and a longer duration of diabetes and certain comorbidities, such as dyslipidemia, chronic kidney disease, peripheral artery disease, obstructive sleep apnea syndrome, etc. [15,26,27,28,29]. Our results showed no statistically significant correlations between the stages of DPN (classified as the TCNSS in subjects) and HTN and, therefore, do not support an association. In agreement with our results, previous reports showed that the presence of HTN was not associated with the progression of DPN. More specifically, the Study of Maastricht [30] and the systematic review of Ross both et al. [31] did not provide evidence for an association between the components of metabolic syndrome and especially HTN and DPN. Baxi et al. also showed no statistically significant association between HTN and DPN [32]. According to the recent review by Lee et al., there is no indication that the monitoring of HTN prevents the development of DPN [33].

The pathogenesis of diabetic neuropathy is complex and involves many factors. Although persistent chronic hyperglycemia has been considered the main cause of microvascular complications, including neuropathy, studies have shown that genetic polymorphisms, hypoinsulinemia or insulin resistance, and hyperinsulinemia may also play a major role [34,35,36].

Hyperglycemia is the main metabolic characteristic of both T2DM and T1DM, and is related to direct axonal damage and demyelination, early metabolic abnormalities of the nerves, an increase in polyol pathway activity resulting in the accumulation of sorbitol and fructose and a decrease in Na^+^/K^+^-ATPase activity, mitochondrial dysregulation, and the activation of inflammation [37,38,39]. Indeed, the best-known treatment for the prevention of neuropathic complications is strict glycemic control. In our population, HbA1c, which represents the mean level of glycemia in the last 2–3 months, was >7% in 52.48% of the participants, and 61.7% of them suffered from moderate to severe DPN (*p*-value 0.08). These results suggest that hyperglycemia played a major role in the development of DPN in our study group.

In addition to hyperglycemia, loss of insulin signaling due to insulin resistance or insulinopenia may also contribute to the development of DPN [13,40]. It is established that there are insulin receptors on the neurons of the peripheral nervous system (PNS) [40,41,42]. Plasma insulin levels within a normal range promote neuronal outgrowth and survival and maintain sensory function by regulating the synthesis of key neuromodulator proteins and peptides [42,43]. Therefore, a decrease in insulin action may have direct effects on neuronal function and metabolism and contribute independently to the development of DPN [43,44].

Insulin resistance, a hallmark of T2DM and obesity, is a clinical condition characterized by the impaired responsiveness of insulin-sensitive tissues to insulin and a compensatory increase in insulin release by β-cells [45]. In healthy subjects or in patients with T2DM/obesity, the development of insulin resistance has been linked to an increased risk for macro- and microvascular complications, independently of hyperglycemia, dyslipidemia, or other cardiovascular risk factors [46,47]. In subjects with impaired glucose tolerance or overt T2DM, Thrainsdottir et al. [48] investigated the association between glucose intolerance and DPN by performing oral glucose tolerance tests and nerve biopsies. In these subjects, DPN was present in 44% and 60% of the individuals with impaired glucose tolerance orT2DM, respectively. Peripheral nerve biopsies showed increased thickening of the endo neural capillary basement membrane and a decreased luminal area during the progression from impaired glucose tolerance to overt T2DM. The authors [49] concluded that capillary microangiopathy and neural hypoperfusion are early mechanisms in the process underlying DPN in T2DM and are due to the presence of insulin resistance. Insulin resistance is also present in T1DM, due, at least in part, to the presence of obesity, chronic hyperglycemia, and the exogenous administration of insulin [49]. In T1DM, insulin resistance at baseline has been reported to be associated with an increased subsequent risk for both macro- and microvascular complications including neuropathy [50].

Insulin resistance is associated with hyperinsulinemia, which has growth-promoting effects; increases the risk for hypoglycemia and high glucose variability, endothelial damage, and proinflammatory and prothrombotic abnormalities; causes hypertension and weight gain, and can also induce or aggravate insulin resistance [51,52]. In T2DM, hyperinsulinemia is present before clinical diagnosis, and during disease progression from prediabetes to overt T2DM [53]. In T2DM, there is a link between reduced insulin signaling and peripheral nervous system (PNS) insulin resistance, resulting in damaged neurite outgrowth [54]. The development of neuronal insulin resistance by insulin administration is supported by studies reporting reduced downstream insulin signaling in vivo in the PNS of insulin-resistant ob/ob mice in response to either IT or IP) injections of insulin [44]. In our study, 18 patients (17.65%) were on insulin treatment alone and 30 patients (29.41%) were on a combination of insulin and oral agents. Of these subjects, 14.58% on insulin alone and 37.5% on a combination of insulin and oral agents had developed moderate/severe DPN. However, this association was not statistically significant (*p* =0.191), suggesting that, in our population, hyperinsulinemia was not associated with DPN development.

In addition to hyperinsulinemia, an association of hypoinsulinemiawith DPN has also been described. In T2DM, the deterioration of insulin secretion and hypoinsulinemia may develop during the progression of the disease due to treatment failure with diet or oral hypoglycemic agents and poor glycemic control [53]. In adults with obesity and T2DM, Partanen et al. [55] investigated the occurrence and risk factors of DPN at five years after the diagnosis of T2DM(mean HbA1c 9.3%), when all subjects were treated with nutritional interventions alone, and after ten years of follow-up (mean HbA1c 9%) when treatment involved a combination of oral hypoglycemic agents and insulin. The results showed that DPN was present at five years of overt diabetes, and its frequency/severity was increased at 10 years, suggesting a cumulative effect of neuropathic factors over time. Postprandial plasma insulin and C-peptide levels at five and ten years were lower than in age/weight-matched controls; furthermore, subjects with DPN had plasma insulin values lower than those without DPN. The authors concluded that hyperglycemia and hypoinsulinemia are strong predictors of both the frequency and severity of DPN in T2DM. In T1DM, hypoinsulinemia is present before clinical diagnosis due to progressive autoimmune β-cell destruction [40,42,56].

Our study showed a statistically significant association between dipping status and DPN, with non-dippers having an almost four times higher risk for moderate/severe DPN than dippers (OR 3.93; 95%CI [1.33–11.64]). This association was independent of HTN and glycemic control. In agreement with our results, Najafi et al. also showed that only dipping status, among all the parameters of ABPM, was a statistically significant risk factor for DPN progression [57].

Non-dipping is one of the most frequent patterns of individuals with diabetes and hypertension [58], and it has been demonstrated that these patients tend to have a higher nocturnal BP [59]. To some extent, non-dipping status is etiologically linked to the underlying cardiovascular autonomic neuropathy in this condition and to the sympathetic nervous system, which paradoxically prevails over the parasympathetic nervous system in these patients during the night [60].

According to Spallone et al. [61,62], the central role in the development of non-dipping/reverse dipping is played by the autonomic dysfunction present in prediabetes and overt T2DM and is characterized by sympathetic overactivity, sympathovagal unbalance, and loss of the circadian autonomic rhythm with sympathetic predominance during the night. Insulin resistance in type 2 diabetes can induce chemoreflex upregulation and baroreflex impairment, thus enhancing sympathetic overactivity. Additional factors are added to the pathogenesis of non-dipping while the disease progresses.

In diabetic autonomic neuropathy, abnormal circadian patterns of blood pressure and sympathovagal balance, with a reduced fall in blood pressure and the prevalence of sympathetic activity during the night, have been described. Orthostatic hypotension and supine hypertension in the advanced stages of autonomic diabetic neuropathy exert a contributory role. Other documented possible contributory factors are fluid redistribution from the extra- to the intravascular compartment in protein uric nephropathy, a compensatory nocturnal-pressure natriuresis in salt-sensitive hypertension in T2DM, and kidney failure, sleep disturbances, and neuropathic pain. According to Mahabala et al., the persistence of daytime BP into the night and the development of a non-dipper status seems to be secondary to the necessity for extended pressure natriuresis [63]. It is reported that, compared with the correlation between daytime sodium excretion and daytime BP, the correlation between nighttime sodium excretion and nocturnal BP is stronger [64]. All these factors act synergistically to disrupt circadian BP patterns and lead to a non-dipping pattern [61].

Non-dipping status or nocturnal hypertension was associated with target organ damage and an increased risk for cardiovascular disease (CVD) [65,66,67,68]. According to Spallone et al. [61], the finding of an abnormal circadian profile in patients with autonomic failure and a decreased vagal tone with simultaneous sympathetic predominance has been postulated as an independent factor of mortality after myocardial infarction. Moreover, nocturnal BP levels may be relevant to target organ damage, since hypertensive patients classified as non-dippers have a significantly higher frequency of stroke or greater left ventricular hypertrophy [62].

Dippers and non-dippers are different in their cardiovascular risk profiles, the severity of the disease, end-organ involvement, and long-term cardiovascular complications.

In our study, the population with diagnosed HTN was treated with one agent or a combination of antihypertensive agents, and the type of antihypertensive therapy did not show any statistically significant association with the stage of DPN. Furthermore, the status of dipping was not previously known, so every patient was on the conventional one-morning-dose treatment. Even though non-dipping has an increased risk for a worse overall prognosis [61,65,69], and the incidence of cardiovascular events is even worse in reverse dippers [70], the 2018 ESC/ESH guidelines do not provide recommendations on when-to-treat [15].

Six main classes of antihypertensive drugs (ACEIs, CCBs, β-blockers, diuretics, ARBs, and α-blockers) have been used worldwide. These drugs are traditionally administered once every morning, based on the results of many clinical trials showing the advantages of morning dosing in reducing the risk of cardiovascular diseases. On the other hand, chronotherapy is defined as a type of purposeful treatment that enhances the effectiveness and tolerance of a drug by choosing the optimal dosing time [71]. In the Heart Outcomes Prevention Evaluation (HOPE) study in 2000, ramipril administered at night demonstrated a decreased CVD risk by controlling nighttime BP; however, there was no direct comparison with daytime dosing in this study [72].Ten years later, the MAPEC study compared morning versus night doses of antihypertensive therapy and concluded that the nighttime dose achieved better overall BP control after a mean follow-up of 5.6 years in 2156 individuals [73]. More recently, the Hygia Chronotherapy Trial has demonstrated that a bedtime hypertension treatment strategy is safe and that the risk for adverse effects is comparable to the more common morning-time treatment strategy [74]. Moreover, in the meta-analysis by Xie et al, a prominent role of evening CCB in lowering blood pressure was reported [75].

Therefore, it may be reasonable to suggest the evening administration of an antihypertensive regimen to treat non-dippers. Furthermore, our study showed that, independently of the DPN stage, the percentage of undiagnosed HTN in patients with DM was 35%.HTN is mainly a silent disease and has a growing prevalence as the population ages. Therefore, undiagnosed HTN can be detected early only when public health systems adapt to national prevention programs. It has been shown that patients with undiagnosed HTN constitute more than 50% of the general population [76,77].Considering that our study population suffered also from DM and was therefore under monitoring and medical treatment for DM and DPN, it was expected that comorbidities, such as HTN, could be detected and controlled to limit the cardiovascular risk of these patients. This assumption could explain why the percentage of undiagnosed HTN was smaller in our patients than in the general population.

Alternatively, the fact that DPN can induce HTN through several mechanisms may also contribute to this 35% of undiagnosed HTN. Mechanisms such as the acceleration of subclinical atherosclerosis and reduction of arterial compliance [78], exacerbation of chronic inflammation, and oxidative stress and abnormal activation of the renin–angiotensin–aldosterone system have been proposed to contribute to the development of HTN in patients with DPN [79]. Another possible mechanism could be that DPN is accompanied by neurologic and parasympathetic/sympathetic damage, which contributes to the increase in BP [50]. Additional mechanisms of DPN-mediated HTN include higher levels of glycosylated end-products, which can also inhibit the release of vasorelaxants, such as nitric oxide and prostacyclin, triggering the release of endothelin and thromboxane A2, and hence lead to vasoconstriction, a decrease in tissue blood flow rates, hypoxia, and metabolic dysregulation [25,80].

Strengths of our study include the well-standardized data collection and the relatively homogenous nature of the study population, residing in distinct geographical locations, including rural and urban areas of Athens, as well as migrant populations. The findings of our study are limited by the small sample size and by the fact that ABPM was performed once. However, the comparison between groups showed clear differences in the statistical analysis. In addition, in our patient groups, there were more normotensive than hypertensive patients. Furthermore, the cross-sectional study design restricts causality inferences, warranting the cautious interpretation of the results.

## 5. Conclusions

In conclusion, our findings do not support an association between DPN and HTN. However, dipping status and female sex were shown to play a role in the pathophysiology of DPN. As 35% of patients with diabetes had undiagnosed HTN and dipping status is a risk factor for DPN, we suggest the use of ABPM both for the diagnosis of HTN and as a tool for the prognosis of DPN development. Early control of HTN with lifestyle changes and antihypertensive medications could contribute to delaying the progression of micro- and macroangiopathy, and therefore protect patients from DPN and improve their quality of life. Therefore, using ABPM once every 6–12 months seems to be a reasonable screening strategy. A better understanding of the mechanism of DPN may contribute to identifying the risk factors involved and enable health providers to set up better prevention programs. However, as our study was a cross-sectional study design, more studies using longitudinal data should be conducted to confirm our results.

## Figures and Tables

**Table 1:** Demographic, clinical, and biochemical characteristics of participants with diabetes. Results are shown as mean with SD in parentheses, unless indicated otherwise.

Characteristics	Total n = 102	Absent/Mild DPN n = 54	Moderate/ Severe DPN n = 48	*p*-Value
Sex, n (%)				0.111 ^1^
Males	64 (62.75)	30 (55.56)	34 (70.83)	
Females	38 (37.25)	24 (44.44)	14 (29.17)	
Age (years)	60.45 (±11.3)	61.91 (±11.37)	58.81 (±11.12)	0.1687 ^2^
BMI (kg/m^2^)	30.37 (±5.94)	28.78 (±4.39)	32.1 (±6.9)	0.0047 ^2^
Duration of HTN (years)	6.88 (±8.7)	8.07 (±9.59)	5.54 (±7.45)	0.1432 ^2^
Duration of DΜ (years)	14.03 (±9.84)	13.94 (±10.08)	14.13 (±9.67)	0.9268 ^2^
DM Type, n (%)				
T1DM	7 (6.86)	2 (3.70)	5 (10.42)	0.181 ^1^
T2DM	95 (93.10)	52 (96.30)	43 (89.58)	
HbA1c (%)	7.65 (±1.76)	7.29 (±1.36)	8.05 (±2.07)	0.0306 ^2^

^1^: *p*-values from Pearson’s X^2^ test; ^2^: *p*-value from two-sample *t*-test; mean (SD): mean (standard deviation); n: number of patients; HTN: hypertension; HbA1c: glycated hemoglobin; DM: diabetes mellitus; BMI: body mass index; T1DM: type 1 diabetes; T2DM: type 2 diabetes; DPN: diabetic peripheral neuropathy.

**Table 2:** Independent-sample *t*-tests for variables of the 24 h ABPM analysis. Results are presented as mean ± SD (in parentheses). SBP: systolic blood pressure; DBP: diastolic blood pressure; 24 h ABPM:24 h ambulatory blood pressure monitoring; DPN: diabetic peripheral neuropathy.

24 h ABPM (mmHg)	Total n = 102	Absent/ Mild DPN n = 54	Moderate/ Severe DPN n = 48	*p*-Value
24 h SBP	123.47 (11.92)	121.96 (12.46)	125.17 (11.17)	0.1767
24 h DBP	71.23 (7.45)	69.59 (7.06)	73.06 (7.52)	0.0181
Daytime SBP	125.4 (11.44)	124.5 (12.02)	126.42 (10.77)	0.4009
Daytime DBP	73.32 (7.21)	72.24 (7.05)	74.54 (7.26)	0.1079
Nighttime SBP	117.39 (16.76)	114.14 (17.5)	121.44 (15.04)	0.0371
Nighttime DBP	64.63 (9.45)	62.33 (8.5)	67.49 (9.88)	0.0085

**Table 3:** Results of Pearson’s X^2^ test for variables of the 24 h ABPM analysis.

24 h ABPM	Total n = 102	Absent/ Mild DPN n = 54	Moderate/ Severe DPN n = 48	*p*-Values
24 h HTN stages, n (%)				
<120/<80	37 (36.27)	21 (38.89)	16 (33.33)	0.854
120–129 and /80–84	31 (30.39)	17 (31.48)	14 (29.17)	
130–139 or /85–89	24 (23.53)	11 (20.37)	13 (27.08)	
>140 or /90	10 (9.8)	5 (9.26)	5 (10.42)	
ABPM patterns, n (%)				0.323
No extra pattern	57 (55.88)	32 (59.26)	25 (52.08)	
WCH	37 (36.27)	16 (29.63)	21 (43.75)	
Masked HTN	7 (6.86)	5 (9.26)	2 (4.17)	
ABPM dipping status, n (%)				0.061
Dippers	36 (37.5)	24 (47.06)	12 (26.67)	
Non-dippers	36 (37.5)	14 (27.45)	22 (48.89)	
Reverse dippers	24 (25)	13 (25.49)	11 (24.44)	

HTN: hypertension, ABPM: ambulatory blood pressure monitoring; DPN: diabetic peripheral neuropathy; WCH: white coat hypertension.

**Table 4:** Odds ratios (OR) and 95% confidence intervals (CI) for the association between stages of DPN and HTN.

	Univariate Model			Multivariate Model		
Variables	OR	95% CI	*p*-Value	OR	95% CI	*p*-Value
Age (in years)	0.98	(0.94–1.01)	0.169	0.98	(0.94–1.02)	0.335
Sex						
Females vs. Males	0.51	(0.23–1.17)	0.113	0.35	(0.14–0.92)	0.033
Smoking						
Yes vs. No	1.75	(0.73–4.21)	0.212			
ΒΜΙ	1.11	(1.02–1.20)	0.008			
HTN (years)	0.96	(0.92–1.01)	0.148			
DM (years)	1.00	(0.96–1.04)	0.926			
HbA1c (%)	1.30	(1.02–1.66)	0.037			
No. of antihypertensives vs. no. medication						
1	0.64	(0.20–2.05)	0.448			
2	0.73	(0.23–2.27)	0.583			
>2	0.5	(0.19–1.33)	0.164			
Type of antidiabetic vs. diet						
Tablets	0.22	(0.02–2.29)	0.206			
Insulin	0.21	(0.18–2.47)	0.215			
Oral agents and Insulin	0.5	(0.04–5.40)	0.568			
ABPM dipping						
Non-dippers vs. dippers	3.14	(1.19–8.24)	0.02	3.93	(1.33–11.64)	0.013
Reverse dippers	1.69	(0.59–4.88)	0.331	1.74	(0.54–5.65)	0.354
ABPM status						
Hypertensives vs. Normotensives	1.43	(0.64–3.20)	0.386			
24 h SBP	1.02	(0.99–1.06)	0.177			
24 h DBP	1.07	(1.00–1.13)	0.022			
Daytime SBP	1.02	(0.98–1.05)	0.397			
Daytime DBP	1.05	(0.99–1.11)	0.11			
Nighttime SBP	1.03	(1.00–1.06)	0.042			
Nighttime DBP	1.06	(1.01–1.12)	1,012			
24 h-Hypertension stages						
<120/<80	Reference category			Reference category		
120–129 and/80–84	1.08	(0.41–2.82)	0.874	0.89	(0.30–2.69)	0.848
130–139 or /85–89	1.55	(0.32–5.32)	0.405	0.95	(0.29–3.14)	0.932
140 or /90	1.31	(0.32–5.32)	0.703	1.18	(0.23–5.99)	0.843

OR: odds ratio, 95% CI: 95% confidence interval, SBP: systolic blood pressure, DBP: diastolic blood pressure, HTN: hypertension, DM: diabetes mellitus, ABPM: ambulatory blood pressure monitoring; SBP: systolic blood pressure; DBP: diastolic blood pressure; HbA1c: glycated hemoglobin.

## Data Availability

No applicable.

## Abbreviations

HTN: hypertension; DPN, diabetic peripheral neuropathy; TCNSS, Toronto Clinical Neuropathy Scale System; CCBs: calcium-channel blockers.
